# Kosten-Erlös-Defizit der ambulanten Versorgung von Kopfplatzwunden in der Notaufnahme

**DOI:** 10.1007/s00104-020-01276-7

**Published:** 2020-09-21

**Authors:** Nils Mühlenfeld, Cora R. Schindler, Jasmina Sterz, Philipp Thönissen, Philipp Störmann, Ingo Marzi, René D. Verboket

**Affiliations:** Goethe Universität Frankfurt, Theodor-Stern-Kai 7, 60590 Frankfurt am Main, Deutschland

## Einleitung

Die Wundversorgung von Kopfplatzwunden im Rahmen der Notfallversorgung gehört als fester Bestandteil zum klinischen Alltag einer Notaufnahme. Die meistens isolierten und im Rahmen eines Bagatelltraumas, eines Sturzes oder einer Körperverletzung ausgelösten Verletzungen der Kopfhaut stellen auch für unerfahrene Chirurgen eine eher geringe technische Herausforderung dar. Allerdings entsteht nicht selten ein Personal- und Zeitproblem, da ein Team aus einem Chirurgen und einer Pflegekraft gleichzeitig beim Patienten gebunden wird. Entsprechend der aktuellen S1-Leitlinie müssen akute Wunden in einem Zeitfenster von unter 6 h versorgt werden [18]. Dies erfordert eine zeitnahe Versorgung, zu der nicht nur die chirurgische Wundversorgung, sondern ebenfalls eine eingehende Anamneseerhebung inklusive Untersuchung zum Ausschluss von Begleitverletzungen mit gegebenenfalls ergänzender radiologischer Bildgebung zum Ausschluss einer knöchernen Mitbeteiligung des Schädels oder eines Schädel-Hirn-Traumas gehört. Ebenso muss die vorherige Lokalanästhesie sowie die Wundreinigung und Desinfektion erfolgen. Liegt außerdem kein ausreichender Tetanusimpfschutz vor, wird nach den Empfehlungen der ständigen Impfkommission (STIKO) eine Auffrischung oder Simultanimpfung durchgeführt [18].

Bei ausgedehnteren Kopfplatzwunden ist die Notfallbehandlung in einer heutzutage zunehmend überfüllten Notaufnahme eine Ressourcenbindung über einen längeren Zeitraum, der nicht selten in deutlich verlängerten Wartezeiten für Folgepatienten resultieren kann. Dies gilt insbesondere bei der Versorgung intoxikierter und/oder dementer Patienten. Auch die regelmäßige Nachsorge zur Prävention einer Infektion oder Nahtdehiszenz ist ein nicht zu vernachlässigender Zeitaufwand für die Krankenhaussprechstunde oder den niedergelassenen Kollegen. Die adäquate Versorgung ist jedoch für das Wohlergehen der Patienten von großer Bedeutung. Leider führt die Vergütung nach einheitlichem Bewertungsmaßstab (EBM) oft zu einer unzureichenden Vergütung der Notfallleistungen und zu einer regelhaften Kostenunterdeckung in den Notaufnahmen [7, 17].

Ziel dieser Arbeit ist es, die Ätiologie von Kopfplatzwunden in einer großen Notaufnahme zu beschreiben und das Kosten-Erlös-Verhältnis der ambulanten Wundversorgung von Kopfverletzungen anhand der Vergütung von Notfallleistungen auf der Kalkulationsgrundlage des EBM zu untersuchen und zu evaluieren, ob die im EBM gegebene Abrechnungsgrundlage mit den auflaufenden Kosten im Einklang steht.

## Studiendesign und Methoden

### Studiendesign und Datenakquise

Die Patientenzahl enthält ausschließlich gesetzlich Versicherte. Selbstzahler, Privatpatienten und berufsgenossenschaftliche Fälle wurden aufgrund anderer Abrechnungsmodalitäten und sehr geringer Fallzahlen ausgeschlossen. Die Daten der insgesamt 724 Fälle wurden in der Zentralen Notaufnahme (ZNA) am Universitätsklinikum Frankfurt am Main im Jahr 2018 erhoben. Mittels einer systematischen Abfrage im Krankenhausinformationssystem (Hospital Information System [HIS]) wurden alle Patienten identifiziert, die in diesem Zeitraum eine Wundversorgung einer Kopfplatzwunde mittels Naht (EBM 02301) erhielten. Fälle mit unklarer Art der Wundversorgung oder unzureichender Dokumentation wurden ausgeschlossen, auch Kinder, bei denen Kopfwunden häufig geklebt werden können, wurden ausgeschlossen. Die Genehmigung der Studie erfolgte unter dem Ethikvotum Nummer 19-491 der Universitätsklinik Frankfurt, die vorliegende Studie folgt den STROBE(Strengthening The Reporting of Observational Studies in Epidemiology)-Richtlinien für Beobachtungsstudien sowie den RECORD(Reporting of studies Conducted using Observational Routinely-collected Data)-Richtlinien für Observationsstudien [4, 6].

### Datenerhebung und statistische Analyse

Die Berechnung der Erlöse erfolgte anhand der einheitlich abgerechneten Notfallpauschale EBM 01210 bei Inanspruchnahme von Montag bis Freitag zwischen 07:00 und 19:00 Uhr und der Notfallpauschale EBM 01212 zwischen 19:00 und 07:00 Uhr des Folgetages, zudem ganztägig an Samstagen, Sonntagen, gesetzlichen Feiertagen und am 24.12. und 31.12. [8, 9]. Die Abrechnung der eigentlichen Wundversorgung erfolgte unter der Pauschale EBM 02301 „Kleinchirurgischer Eingriff II und/oder primäre Wundversorgung mittels Naht“. Mit dieser Pauschale werden neben der Hautnaht unter Lokalanästhesie z. B. die Entfernung eines unter der Hautoberfläche gelegenen Fremdkörpers, die Deckung eines Hautdefektes sowie die Eröffnung eines subkutanen Abszess abgerechnet [10].

### Kostenkalkulation

Die Fallkostenkalkulation erfolgte in Zusammenarbeit mit dem operativen Controlling. Es wurde eine Aufschlüsselung der anfallenden Einzelposten erstellt (Tab. 1). Die Berechnung der Personalkosten erfolgte getrennt für Ärzte und Pflegepersonal nach ihren spezifischen Entgelten. Die Minutenkosten für das Personal wurden auf Basis der ab 20.02.2018 geltenden Entgelttabelle nach Tarifvertrag für Ärztinnen und Ärzte an den hessischen Universitätskliniken (TV-Ärzte Hessen 2018) berechnet. Bei einer 42-Stunden-Woche wurden für den Notaufnahmedienst (Durchschnittsgehalt aus 1./4./7. Weiterbildungsjahr) die Minutenentgelte und der Arbeitgeberanteil für die Lohnnebenkosten im Jahr 2018 mit 19,375 % berechnet. In den Lohnnebenkosten wurden die Renten‑, Arbeitslosen‑, Kranken- und Pflegeversicherung für einen imaginären Arbeitnehmer mit Lohnsteuerklasse 1, gesetzlicher Krankenversicherung und ohne Kinder erfasst. Die Nettojahresarbeitsstunden wurden unter der Berücksichtigung einer Ausfallzeit von 2,1 % für Ärzte berechnet [11]. So konnte ein Entgelt von 0,62 €/min festgestellt werden. Der Minutenpreis des Pflegepersonals wurde anhand des Tarifvertrags für die Johann-Wolfgang-Goethe-Universität Frankfurt am Main (TV-G-U) in der Fassung des Änderungstarifvertrages vom 11.09.2017 (Entgelttabelle für Pflegekräfte, gültig ab 01.01.2018) für 38,5 Wochenstunden ermittelt. Als Referenz diente die vorwiegend beteiligte Entgeltgruppe P8. Die durchschnittlichen Personalkosten für die Stufen 3 und 4 wurden gemittelt. Der Arbeitgeberanteil der Lohnnebenkosten wurde entsprechend berechnet und eine Ausfallzeit von 6,85 % berücksichtigt [11]. Auf diese Weise wurde ein Minutenentgeld von 0,42 €/min errechnet.

**Table 1:** 

Verbrauchsmaterial	Anzahl	Kosten
Wundversorgungsset, steril (Einmalinstrumente)	1	9,30 €
Tupfer, steril (3er Pack)	1	0,21 €
Abdecktuch, steril	1	0,46 €
Lochtuch, steril	1	0,57 €
Haube	1	0,05 €
Mundschutz	2	0,07 €
Handschuhe, steril	1	0,31 €
Nahtmaterial	1	3,91 €
Einmalskalpell, steril	1	3,50 €
Spritze 10 ml, steril	2	0,08 €
Kanüle, steril	2	0,02 €
Kompressen, steril	2	0,06 €
Mullbinde	1	0,07 €
NaCl 0,9 % 10 ml	2	0,06 €
Desinfektion (z. B. Octenisept^©^ (Octenidindihydrochlorid), Braunol^©^ (PVP-Iod))	300 ml	1,78 €
Lokalanästhetikum (z. B. Mecain^©^ (Mepivacain))	5 ml	0,29 €
*Summe Material: 20,74* *€*		
*Arzt*		
Durchschnittliche Dauer der Aufnahme und Wundversorgung (min)	18,4	*11,41* *€*
*Pflege*		
Durchschnittliche Dauer der Triage, Vorbereitung, Assistenz und Nachbereitung (min)	28,5	*11,97* *€*
*Summe Personal: 23,38* *€*		
*Gesamtsumme: 44,12*		

Die Kosten für Verbrauchsmaterial, Desinfektionsmittel und Lokalanästhetikum und weiteres Nötige (Tab. 1) wurden über unseren zentralen Einkauf bzw. die Klinikapotheke erhoben, hier sind die Einkaufspreise aufgeführt.

Die Wundversorgung wurde für alle Patienten entsprechend der S1-Leitlinie durchgeführt und die durchschnittliche Behandlungsdauer einer Standardwundversorgung mittels einschichtiger Hautnaht zugrunde gelegt, hierzu erfolgte eine Messung der durchschnittlich notwendigen Zeit für die Wundversorgung auf Seiten der Pflege und des ärztlichen Dienstes. Etwaige zusätzliche Leistungen der Radiologie oder des Labors wurden nicht berücksichtigt.

Die Kosten der einzelnen Faktoren wurden zu einem Gesamtkostenbetrag addiert und anhand der prozentualen Fallverteilung mit der Gesamtvergütung nach EBM verglichen (Tab. 2).

**Table 2:** 

**Kosten**			
Personalkosten			23,38 €
Materialkosten			20,74 €
*Summe:*			*44,12* *€*
**Erlöse**			
EBM 02301 nach GOÄ Hessen	13,96 €	724 Pat	–
EBM 01210 nach GOÄ Hessen	12,99 €	202 Pat	–
EBM 01212 nach GOÄ Hessen	21,10 €	522 Pat	–
(EBM 02301 * 724 + [EBM 01210 * 202 + EBM 01212 * 522])/724		Ø	–
*Summe:*			*32,80* *€*
*Gesamterlös pro Fall:*			*−11,32* *€*
*Kosten-Erlös-Defizit*			*−8195,68* *€*

## Ergebnisse

Über das HIS konnten 724 Patienten, welche zwischen dem 01.01.2018 und dem 31.12.2018 mit einer Kopfplatzwunde in der Notaufnahme vorstellig wurden, identifiziert werden. In allen Fällen erfolgte eine Wundversorgung mittels Naht. Patienten mit kleineren Wunden, welche nicht chirurgisch wundversorgt werden mussten, wurden im Vorfeld aussortiert. Es zeigte sich eine vornehmlich männliche (68,2 %, 495/724) Kohorte jüngeren Alters (42 ± 21,5 Jahre).

### Vorstellungszeiten

**Figure 1:**
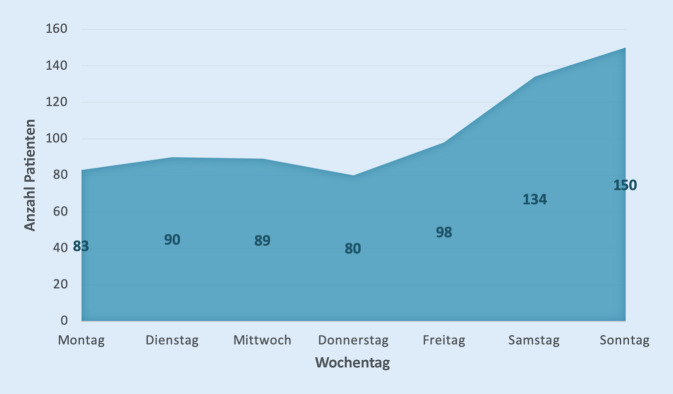

Es zeigte sich eine gehäufte Vorstellung von Patienten mit Kopfplatzwunden am Wochenende (39,2 %, 284/724) Die meisten Vorstellungen erfolgten am Sonntag (20,7 %, 150/724). Knapp über die Hälfte (50,1 %, 369/724) wurde dabei während der Nachtzeit zwischen 19:00 und 7:00 Uhr behandelt, die meisten zwischen 20:00 Uhr und 21:00 (8,7 %, 63/724). Die Patientenanzahl an den jeweiligen Tagen bzw. zu den jeweiligen Uhrzeiten können den Abb. 1 und 2 entnommen werden.

**Figure 2:**
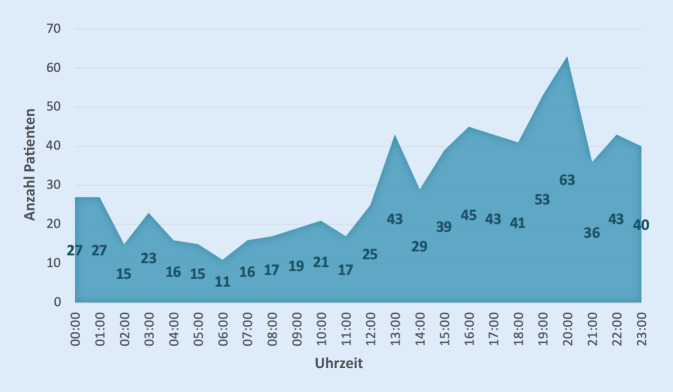

Im untersuchten Zeitraum stellten sich 202 (27,9 %) Patienten innerhalb der Regelarbeitszeiten von Montag bis Freitag zwischen 7:00 und 19:00 Uhr vor (EBM 01210) und wurden mit 120 Punkten und somit mit 12,99 € nach der Gebührenverordnung für Ärzte (GOÄ) in Hessen abgerechnet.

Insgesamt 522 Patienten (72,1 %) stellten sich dementsprechend nachts und/oder am Wochenende vor (EBM 01212) und wurden mit 195 Punkten, entsprechend 21,10 € (GOÄ Hessen) bewertet. Für die Durchführung einer Wundversorgung wurden nach EBM 02310 13,96 € (129 Punkte, GOÄ Hessen) abgerechnet.

### Erlöse

Der Gesamterlös für eine Wundversorgung tagsüber unter der Woche berechnet sich auf 26,95 € und pro nächtlichen Notfall bzw. am Wochenende oder an Feiertagen auf 35,06 €. Bei 202 Fällen nach EBM 01210 entspricht das 5443,90 € für die Behandlung von Kopfplatzwunden unter der Woche. Die 522 nachts und am Wochenende (EBM 01212) durchgeführten Behandlungen erzielten Einnahmen von 18.301,32 €. Somit errechnet sich ein Gesamterlös von 23.745,22 € für den retrospektiv untersuchten Zeitraum von Januar 2018 bis Dezember 2018 (12 Monate).

### Kosten

Die Kostenkalkulation für Personal erfolgt wie im Absatz Material und Methoden beschrieben und in Tab. 1 aufgeführt. Für die Personalkosten der Ärzte im Dienst wurden 0,624 €/min berechnet. Für das Pflegepersonal wurde ein Minutenentgelt von 0,412 € berücksichtigt. Der durchschnittliche Zeitaufwand des ärztlichen Personals für eine Wundversorgung inklusive der eingehenden Anamnese, Untersuchung, Beratung bzw. Aufklärung, Behandlung sowie die abschließende Erstellung eines Arztbriefs und Ausstellen von Rezepten und ggf. Krankmeldung belaufen sich auf durchschnittlich 18,4 min. Die assistierende Pflege hat einen Zeitaufwand von 28,5 min durch die vorherige Triage, Patienten- und Materialvorbereitung sowie die Verbandanlage und Nachbereitung des Behandlungsraumes. Damit entstehen 23,38 € Gesamtpersonalkosten, anteilig 11,41 € für die ärztliche und 11,97 € für die pflegerische Leistung.

Die Verbrauchsmaterialien kosten pro Wundversorgung 20,74 € (Tab. 1). Kosten für Reinigung, Verwaltung und Geräte- und andere Vorhaltekosten wurden nicht berechnet.

### Kosten-Erlös-Verhältnis

Es entstehen pro versorgten Patienten Gesamtkosten von 44,12 €. Dem gegenübergestellt wurde die durchschnittliche Vergütung von 32,80 € pro Fall.

Nach Abzug der Kosten von den Erlösen gegenüber den Krankkassen ergibt sich ein Defizit von 11,32 € pro Fall. Bei einem Aufkommen von 742 Patienten mit einer Kopfplatzwunde im Jahr 2018 entspricht dies einem Jahresdefizit von 8195,68 € (Tab. 2).

### Traumamechanismen

Die häufigste Traumaursache für eine Kopfplatzwunde war in unserer Kohorte das Bagatelltrauma (30,2 %, 219/724), also ein Anpralltrauma des Kopfes an stumpfen Gegenständen wie z. B. an Fensterkanten, Möbeln oder an aus geringer Höhe auf den Kopf herabfallende kleinere Gegenstände.

**Figure 3:**
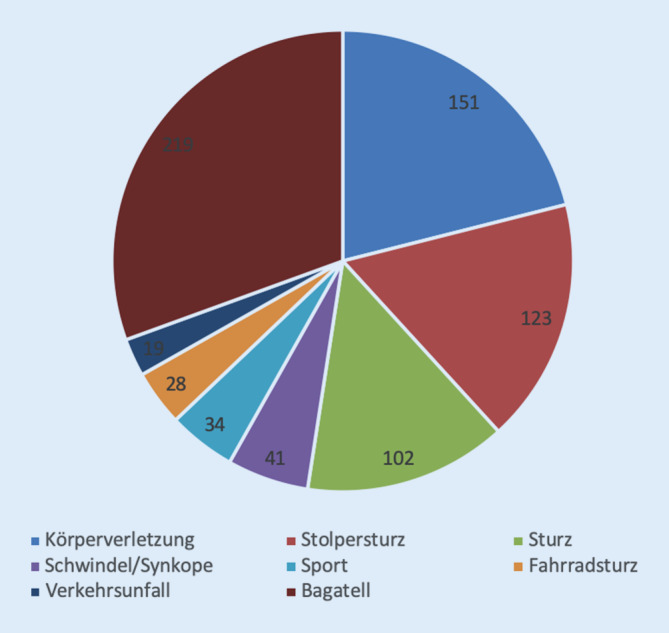

Ein nicht zu vernachlässigender Anteil unserer Patienten stellten sich nach einer körperlichen Auseinandersetzung oder Körperverletzung vor (20,9 %, 151/724). Der dritthäufigste ursächliche Traumamechanismus in unserer Kohorte war der Stolpersturz mit konsekutivem Kopfanprall mit 17,0 % (123/724) aller Vorstellungen (Abb. 3).

### Alkohol und Kopfplatzwunden

Unter den 724 Patienten, welche 2018 mit einer Kopfplatzwunde in unserer Zentralen Notaufnahme vorstellig wurden, erfolgte bei 268 (37,0 %) die Dokumentation einer Alkoholintoxikation.

## Diskussion

Bei einer nach §108 SGB V gegebenen Verpflichtung der zugelassenen Krankenhäuser Notfallversorgung zu betreiben, ergibt sich anhand des Beispiels einer Gegenüberstellung von Erlösen und Kosten der ambulanten chirurgischen Versorgung von Kopfplatzwunden in der Vergütung nach EBM ein deutliches Defizit. Bereits seit Jahren ist das bekannte Defizit zwischen den deutlich zu hohen Kosten der Notfallversorgung in Deutschlands Krankenhäusern Thema wichtiger Diskussionen. Dabei muss einerseits die geringe Vergütung der Notfallversorgung, aber auch das zunehmend hohe Patientenaufkommen in einer für jedermann jederzeit frei zugänglichen Notaufnahme thematisiert werden. In einem System, welches von zunehmender Knappheit der verfügbaren Ressourcen geprägt ist, wird auch die Organisation der stetig zunehmenden Nachfrage nach medizinischer Notfallbehandlung sowie dessen Finanzierbarkeit zunehmend schwierig. Um keine Abstriche in der Verfügbarkeit und Qualität der Versorgung zu befürchten, muss bei Zunahme der Behandlungsfälle die Wirtschaftlichkeit der einzelnen Leistungen analysiert und optimiert werden.

### Regredienz von Notfällen im kassenärztlichen Bereitschaftsdienst

Der starken Regredienz an Notfällen im kassenärztlichen Bereitschaftsdienst von 2009 bis 2015 um 15 % steht die stetig steigende Zahl der ambulanten Notfälle im Krankenhaus gegenüber. Dabei zeigte sich ein Anstieg der Fälle von 6 Mio. im Jahr 2009 auf über 8,5 Mio. im Jahr 2015 [17]. Diese Steigerung um 42 % verdeutlicht die Verschiebung der Notfallvorstellung in die Notaufnahme [1, 13, 17]. Die Frage der Finanzierbarkeit der Notaufnahmen wird damit zunehmend wichtig. Um dieser gerecht zu werden und um Vergütungsdefizite offen zu legen, werden einzelne Leistungen anhand ihrer Wirtschaftlichkeit analysiert [7]. Bisher durchgeführte Untersuchungen konzentrierten sich vor allem auf besondere Krankheitsbilder, polytraumatisierte Patienten oder bildeten die Gesamtkosten der ambulanten Notfallversorgung ab [12, 21, 22, 25–27]. Für die allgemeine Notfallversorgung konnte ein fallbezogener Erlös von 32 € bei durchschnittlichen Kosten von 120 € aufgezeigt werden. Daraus resultierte ein Fehlbetrag von 88 € pro Fall [14–16, 24]. Anhand dieser Arbeit konnte am Beispiel der Versorgung einer einfachen Kopfplatzwunde, die in jeder Klinik häufig durchgeführt wird, ein Vergütungsdefizit von 11,32 € gezeigt werden.

Rund die Hälfte (52 %) der 2 Mio. Verletzten, welche sich in den Notaufnahmen der öffentlichen Krankenhäuser vorstellt, verbleibt ambulant. Dabei erfolgt die Abrechnung von 80 % der Fälle über das EBM-System mittels GKV [18].

Nach dem Projektbericht der Kassenärztlichen Bundesvereinigung zur Notfallversorgung in Deutschland vom April 2018 steigt die Anzahl der ambulanten Notfälle am Wochenende um etwa 39 %. Die meisten ambulanten Notfallbehandlungen werden werktags in der Zeit zwischen 18 und 20 Uhr durchgeführt und am Wochenende zwischen 10 und 12 Uhr [17]. Wie erwartet belegt auch diese Studie die vermehrte Vorstellung von Notfallpatienten mit Kopfplatzwunden außerhalb der regulären Öffnungszeiten der niedergelassenen Ärzte, nämlich hauptsächliche am Wochenende, besonders sonntags, sowie zwischen 18 und 22 Uhr. Insgesamt stellten sich lediglich 27,9 % der Patienten Montag bis Freitag zwischen 7 und 19 Uhr und damit 72,1 % entweder nachts oder am Wochenende vor.

### Probleme durch die Abrechnung nach EBM

Inhalt und Umfang anrechnungsfähiger Leistungen im Bereich der vertragsärztlichen Versorgung werden auf Kalkulationsgrundlage der betriebswirtschaftlichen Aufwandserfassung im niedergelassenen Bereich durch den EBM geregelt [5]. Natürlich sind die Strukturen in einem Krankenhaus nicht mit denen des niedergelassenen Bereichs vergleichbar. Wie in dieser Studie gezeigt, kann der Standardfall der ambulanten chirurgischen Wundversorgung von Kopfplatzwunden durch die EBM-Pauschalen keine ausreichende Kostendeckung garantieren. In dieser Studie errechnete sich für 742 behandelte Patienten ein relevanter Fehlbetrag von 8195,68 € pro Jahr. Dieser ergibt sich aus dem hohen personellen Aufwand, besonders bei alkoholisierten Patienten, sowie dem hohen Anteil an Materialkosten. Hinzukommen die der Einfachheit halber nicht mit eingerechneten Kosten für Verwaltungs‑/Aufnahmepersonal, Abrechnung, Reinigung, Sicherheitspersonal sowie die Zuschläge für Nacharbeit und anfallenden Freizeitausgleich des Personals. Jedoch werden auch diese nicht durch die EBM-Pauschale 02301 abgedeckt. Es ist also von einem reell deutlich höheren Fehlbetrag auszugehen. Durch die aktuelle Vergütung, welche auf die Struktur der Praxis ausgerichtet ist, entsteht in den Notaufnahmen ein klares Defizit [3, 17].

### Mögliche Lösungsansätze

Die Anpassung der Maßstäbe, der Bewertung der einzelnen Leistungen der Notfallversorgung, könnte ein Lösungsansatz darstellen. Dies ist im Fall der Versorgung von Kopfplatzwunden, wie durch diese Studie belegt, definitiv notwendig. Eine Aufwertung würde eine suffiziente Kostendeckung sicherstellen. Ein weiterer Lösungsansatz wäre eine Zusatzziffer für Leistungen, welche im Falle einer Versorgung durch Nichtvertragsärzte zum Tragen kommt. Dies würde keine Änderung des EBM-Vergütungssystems notwendig machen und somit die Abrechnung der Vertragsärzte nicht beeinflussen.

Gemäß § 75 Abs. 1 Satz 2 SGB (Sozialgesetzbuch) V ist die Kassenärztliche Vereinigung verpflichtet, die Versorgung durch Vertragsärzte im Notfalldienst rund um die Uhr sicherzustellen. Der Versicherte wird jedoch nicht dazu verpflichtet, diese Art der Notfallversorgung in Anspruch zu nehmen. Patienten haben nach § 76 Abs. 1 SGB V das Recht, im Falle eines Notfalls auch Krankenhäuser zu konsultieren [17]. Der Ärztliche Bereitschaftsdienst (ÄBD) könnte eine Entlastung der Notaufnahmen deutscher Krankenhäuser bewirken. Als wichtige Stelle des Erstkontakts besteht hier das Potenzial, durch eine bessere und überlegtere Patientensteuerung zu einer besseren und ökonomischeren Notfallversorgung beizutragen [2, 17].

### Großer Anteil an Patienten mit Alkoholeinfluss

Einen nicht zu vernachlässigenden Anteil der in der Notaufnahme mit einer Kopfplatzwunde vorstelligen Fälle stellen unter Alkoholeinfluss stehende Patienten dar; in unserer Kohorte lag dieser Anteil bei 37,0 %. Diese Patienten sind bekanntermaßen schwer einschätzbar, da die Menge und der Zeitpunkt der Einnahme meist unbekannt bleiben. Bei steigenden Fallzahlen an alkoholisierten Patienten in deutschen Notaufnahmen werden Ärzte und das Pflegepersonal vor allem nachts und am Wochenende zunehmend gefordert und auch zeitlich gebunden [23]. Die Alkoholintoxikation ist dabei ein eigenständiges Krankheitsbild, welches ein gut ausgebildetes Team fordert und einen hohen Mehraufwand bedeutet. Nicht nur die Anamnese und Untersuchung, sondern auch die Versorgung ist in dieser Patientengruppe deutlich erschwert. Diese erschwerenden Umstände, die nicht nur eine vermehrte Ressourcenbindung, sondern auch einen deutlich vergrößerten Zeitaufwand bedeuten, werden in der aktuellen Vergütung der Notfallversorgung nicht abgebildet und stellen einen zusätzlichen relevanten Faktor für Kostendefizite für die Notaufnahme dar [19, 20].

### Limitationen

Im komplexen Umfeld einer Klinik konnten nicht alle Faktoren erfasst werden. Wie bereits erwähnt, wurden Kosten für Verwaltungs‑/Aufnahmepersonal, Reinigung, Abrechnung sowie die Zuschläge für Nacharbeit und anfallenden Freizeitausgleich nicht erfasst. Vorhaltekosten für Personal und Investitionskosten wurden ebenfalls nicht berücksichtigt. Jedoch kommt es in dieser Erhebung auch ohne die zuvor genannten Faktoren zu einer Minderdeckung bei der einfachen Versorgung einer Kopfplatzwunde. Es zeigte sich zudem bei der Auswertung der Daten nur ein niedriger Anteil an privat (14) und berufsgenossenschaftlich (23) abgerechneten Patienten in unserem Kollektiv von 2018. Die beispielhafte Versorgung eines privaten Patienten am Samstagnachmittag, welche mit einem 2,3-fach berechneten Satz nach GOÄ einen Erlös von 35,78 € erzielt, ist daher in Bezug auf den Gesamtfehlbetrag vernachlässigbar. Gerade bei den privat versicherten Patienten scheinen andere Strukturen als die Notaufnahme, z. B. der privat ärztliche Notdienst, primäre Anlaufstrukturen zu sein.

## Fazit für die Praxis

Die bisherige Abrechnung der ambulanten Wundversorgung in den Notaufnahmen der Krankhäuser durch den einheitlichen Bewertungsmaßstab ist höchst unzureichend. Eine Alternative oder Erweiterung des Vergütungssystems für den Krankhaussektor bzw. die Einführung einer konsequenteren Patientensteuerung scheinen unumgänglich, um der defizitären Finanzstruktur der Notaufnahmen entgegenzuwirken.
